# Mechanisms of nurses’ AI use intention formation in Sichuan, Yunnan, and Beijing, China: mediating effects of AI literacy via self-efficacy-to-attitude pathways

**DOI:** 10.3389/fpubh.2025.1622802

**Published:** 2025-07-10

**Authors:** Qin Zeng, Xi Huang, Jun Zhu, Shaoyu Su, Yanling Hu, Xiujuan Zhang

**Affiliations:** ^1^Department of Pediatrics Nursing, West China Second University Hospital, Sichuan University, Chengdu, China; ^2^Key Laboratory of Birth Defects and Related Diseases of Women and Children, Sichuan University, Ministry of Education, Chengdu, China; ^3^Department of Neonatology Nursing, West China Second University Hospital, Sichuan University, Chengdu, China; ^4^Department of Pediatrics, National Office for Maternal and Child Health Surveillance of China, National Center for Birth Defect Surveillance of China, West China Second University Hospital, Chengdu, China

**Keywords:** artificial intelligence, literacy, self-efficacy, attitude, usage intention, nursing, China

## Abstract

**Aim:**

This study aimed to explore the formation mechanism of artificial intelligence (AI) usage intention among nurses in public hospitals in Beijing, Sichuan, and Yunnan, China, analyzing the influence of AI literacy on usage intention through AI self-efficacy and general attitudes.

**Methods:**

A multi-center cross-sectional design was adopted, surveying 901 registered nurses via the Wenjuanxing platform from December 26, 2024, to February 25, 2025, with 878 valid questionnaires returned (effective rate 97.45%). Data were collected using the AI Literacy Scale (AILS), General Attitudes toward AI Scale (GAAIS), AI Self-Efficacy Scale (AISES), and AI Usage Intention Scale. Descriptive statistics, correlation analysis, and structural equation modeling (SEM) analysis were conducted using SPSS 26.0 and AMOS 26.0, with case weighting adjustments based on the total number of nurses in each region.

**Results:**

Of the respondents, females accounted for 94.08%, those aged 40 and below accounted for 84.03%, and only 14.24% of nurses had received AI training. The average scores for GAAIS, AILS, and AISES were 69.33 ± 10.31, 56.27 ± 8.60, and 107.92 ± 22.35, respectively, with higher scores observed among nurses with master’s degrees or above, preceptors, and those in Beijing. GAAIS showed strong positive correlations with AILS (r = 0.549), GAAIS with AISES (r = 0.567), and AILS with AISES (r = 0.684, *p* < 0.001), and AI usage intention was closely correlated with all three (*p* < 0.001). Structural equation modeling analysis indicated that the direct effect of AI literacy on usage intention accounted for 30.51%, with indirect effects through AI self-efficacy (21.41%) and general attitudes (14.58%), resulting in a total effect of 0.967 (*p* < 0.001).

**Conclusion:**

AI literacy effectively promotes nurses’ AI usage intention by enhancing their self-efficacy and improving their attitudes toward AI, with self-efficacy being particularly crucial. This mechanism, combining both direct and indirect effects, suggests that enhancing confidence and knowledge is key to promoting AI acceptance. Given the low training participation rate (14.24%) and regional disparities (Beijing outperforming Yunnan), it is recommended that hospitals implement systematic AI training, prioritizing groups with low training exposure and underdeveloped regions, while simultaneously improving attitudes through promotional activities to advance the widespread adoption of AI in nursing and elevate patient care standards.

## 1 Introduction

With the rapid development of artificial intelligence (AI) technology in healthcare, its potential to enhance nursing quality and efficiency has become increasingly evident. Systematic and scoping reviews indicate that AI technologies, such as intelligent diagnostic systems, nursing robots, and electronic health record management, have been widely applied in clinical practice, providing nurses with efficient decision-making support and patient management tools (1–4). For instance, recent studies have proposed a predictive framework for IoT-based smart healthcare systems, leveraging predictive analytics to accurately estimate child mortality rates, providing real-time intervention support in resource-limited regions (5). Furthermore, AI technologies can optimize nursing processes and improve patient outcomes (6), particularly in the security of smart IoT devices, such as intrusion detection systems for people with disabilities, which have shown significant application potential (7). However, these reviews consistently highlight challenges faced by nurses, including low technology acceptance, insufficient AI-related knowledge, and a lack of confidence, which significantly hinder the adoption of AI in nursing practice (2, 4). As a core component of the healthcare system, nurses play a critical role in the implementation and application of AI technologies, with their attitudes and willingness to use AI directly impacting its effective integration into clinical practice (8). Therefore, systematically exploring the factors influencing nurses’ AI adoption intentions and their underlying mechanisms is essential for promoting AI applications in nursing and providing theoretical and practical insights to enhance care quality.

In China, AI technologies are gradually being integrated into the healthcare system, particularly in smart medical devices and data-driven nursing management (9). Comprehensive literature suggests that research on AI applications in nursing is predominantly focused on Western countries, with limited attention to the unique characteristics of China’ s healthcare system, cultural context, and technological resources (2, 4). A cross-sectional study of Chinese nursing professionals found that only 57% of respondents had a basic understanding of AI, 64.7% had little knowledge of its specific applications in nursing, and 13.4% were completely unaware of AI in nursing, despite generally holding positive attitudes toward AI (8). Furthermore, significant disparities in medical resources and technological levels exist across different regions in China. For instance, Beijing, as a technologically advanced municipality, boasts sophisticated medical facilities and greater opportunities for AI technology adoption, whereas Sichuan and Yunnan, constrained by economic and technological limitations, offer nurses fewer opportunities to engage with AI (3, 9). Recent studies have analyzed infant mortality rates in Pakistan and Ethiopia using data mining models, revealing the impact of regional differences on nursing data analysis (10), which resonates to some extent with the regional challenges faced by Chinese nurses. However, existing research rarely addresses regional variations in Chinese nurses’ willingness to adopt AI, particularly in applications for child health prediction and nursing data management (5, 10).

This study constructs a theoretical framework based on the Technology Acceptance Model (TAM) and the Theory of Planned Behavior (TPB) to explore the formation mechanism of nurses’ AI usage intention. The Technology Acceptance Model (TAM) posits that perceived usefulness and perceived ease of use influence behavioral intentions through attitudes, while the Theory of Planned Behavior (TPB) further emphasizes the critical role of self-efficacy (perceived behavioral control) in shaping behavioral intentions (11–13). Extensive research indicates that individuals’ attitudes toward technology reflect their overall cognitive and emotional orientations, while self-efficacy measures their confidence in using technology, which is particularly crucial in AI technology applications (14, 15). Systematic reviews further suggest that positive AI attitudes and high self-efficacy significantly enhance technology adoption intentions (2, 4). Moreover, AI literacy, as a foundational variable, may indirectly influence adoption intentions by enhancing attitudes and self-efficacy (8, 16). Recent studies have also explored the application of multimodal generative AI and autoregressive large language models (LLMs) in human motion understanding and generation, demonstrating how AI can enhance the contextual relevance of nursing-related tasks through semantic alignment (17). This offers new perspectives for improving nurses’ AI literacy. However, existing studies have rarely systematically explored the mediating mechanisms among AI literacy, attitudes, and self-efficacy, particularly within the context of Chinese nurses.

Research on AI applications in nursing has made some progress, but systematic reviews reveal several shortcomings (1, 2, 4). Firstly, most studies focus on single variables (such as attitudes or self-efficacy), lacking systematic analysis of the mediating mechanisms among AI literacy, attitudes, and self-efficacy. Second, empirical studies targeting Chinese nurses are scarce, especially those considering regional differences (e.g., Sichuan, Yunnan, and Beijing). Finally, research on AI applications in nursing predominantly centers on Western countries, with the unique characteristics of Chinese nurses—such as healthcare system, cultural background, and technological resources—remaining underexplored. Thus, investigating how AI literacy affects AI usage intention through self-efficacy and attitude pathways among nurses in Sichuan, Yunnan, and Beijing holds significant research value. Recent studies have proposed a lightweight multistage holographic attention network for image super-resolution, demonstrating AI’s optimization potential in resource-constrained environments (18), which is highly relevant to the current state of nursing technology applications in some regions of China. Additionally, the use of unified large language models to detect misinformation in low-resource languages (19) offers new insights for information management in nursing, indirectly supporting nurses’ trust and adoption of AI tools.

This study targets nurses in Sichuan, Yunnan, and Beijing, China, to examine the mechanisms by which AI literacy influences AI usage intention through self-efficacy and general AI attitudes. We propose the following hypothetical: (1) H1: AI literacy directly affects usage intention by enhancing self-efficacy; (2) H2: AI literacy indirectly influences usage intention by improving general AI attitudes; (3) H3: AI literacy further impacts attitudes via self-efficacy, subsequently affecting usage intention. Employing a cross-sectional survey method, this study uses multidimensional scales (General Attitudes toward AI Scale, AI Literacy Scale, AI Self-Efficacy Scale) to assess nurses’ related variables, with structural equation modeling (SEM) applied to validate these mediating pathways.

**Figure 1:**
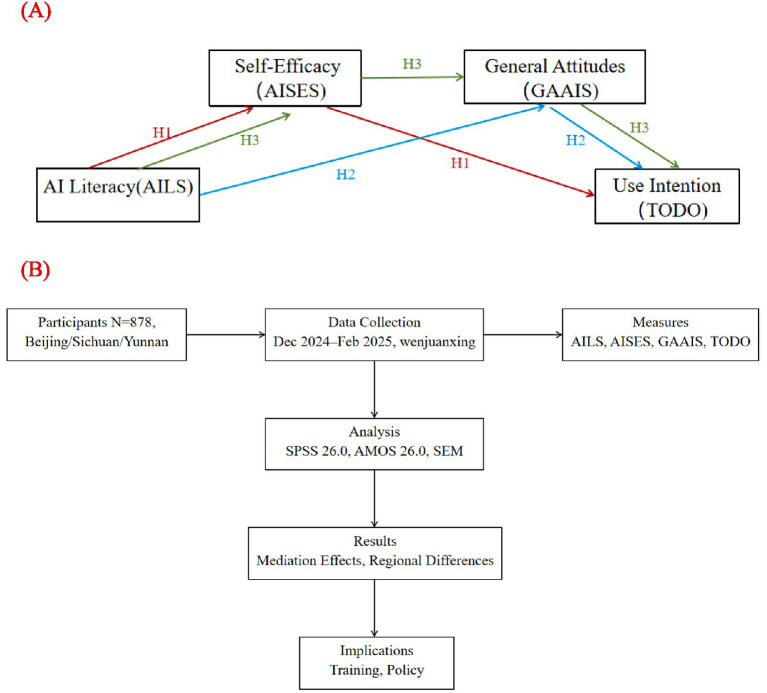
Framework of the study. **(A)** Theoretical model illustrating the mediating pathways from AI literacy (AILS) to AI use intention (TODO) via AI self-efficacy (AISES) and general attitudes (GAAIS). **(B)** Research process, including participant selection, data collection, measurement tools, and analytical methods.

Theoretically, this study validates the mediating effects of AI literacy on usage intention through self-efficacy and attitude pathways, enriching the application of TAM and TPB in nursing. Existing reviews indicate that the formation mechanisms of AI adoption intentions remain underexplored in non-Western contexts (2, 4). This study aims to fill the gap in research on regional differences and mediating mechanisms among Chinese nurses. Practically, the findings can provide a basis for hospital administrators to develop targeted AI training programs. For instance, in regions with limited technological resources, such as Sichuan and Yunnan, enhancing AI literacy and self-efficacy can boost adoption intentions, while in technologically advanced Beijing, optimizing AI attitude training can facilitate technology implementation (3, 20). Moreover, the findings offer policymakers guidance to promote the widespread application of AI in nursing practice, ultimately improving nursing quality and patient care outcomes (Figure 1).

## 2 Participants and methods

### 2.1 Study participants

This study targeted registered nurses from public hospitals in Beijing, Sichuan, and Yunnan, China, aiming to explore the roles of AI literacy, self-efficacy, and attitudes in the formation mechanism of nurses’ artificial intelligence (AI) usage intention, with a particular focus on the mediating effect of AI literacy through the pathway of self-efficacy to attitudes. Considering the potential demand for AI technologies in nursing services, eligible registered nurses were recruited through the Chinese Nursing Association. A preliminary literature review indicated insufficient prior data on AI usage intention and related mediating mechanisms among nurses in these regions to provide an *a priori* proportion. Thus, the expected proportion of nurses’ attitudes and usage intention toward AI was assumed to be 50% (*p* = 0.50). Based on the sample size calculation formula for cross-sectional studies, $n=\frac{Z^{2}p\;(1-p)}{E^{2}}\;$ with a margin of error set at 5% (E = 0.05) and a confidence level of 95% (Z = 1.96), the minimum required sample size for simple random sampling was calculated as 385 participants. Accounting for a 15% non-response rate (based on empirical data from similar studies), the adjusted sample size was 453 participants (385 ÷ 0.85). According to the China Statistical Yearbook 2023, the number of nurses in public hospitals in Beijing, Sichuan, and Yunnan was 126,111, 318,267, and 189,852, respectively. Based on proportional weighting $\left(w_{\text{Province}}=\frac{\text{Nurse in Province}}{\text{Total Nurse in}\;\mathrm{All}\;\text{Province}}\right)$ , the sample required at least 90 participants from Beijing $\left(x1=453\times \frac{126111}{126111+318267+189852}\right)$ , 227 from Sichuan $\left(x2=453\times \frac{318267}{126111+318267+189852}\right)$ , and 136 from Yunnan $(x3=453\times \frac{189852}{126111+318267+189852}).$ Due to the use of convenience sampling within hospitals in three provinces, there may be intra-cluster correlations among nurses from the same province in terms of cognition or attitudes. Therefore, a clustered sampling structure was considered in the sample design. Using provinces (Beijing, Sichuan, and Yunnan) as the clustering units, the average sample size per province was 151. Based on commonly reported intraclass correlation coefficient (ICC) values in social science and health research (typically ranging from 0.01 to 0.05) (21), an ICC of 0.03 was conservatively assumed. Accordingly, the design effect (DEFF) was estimated as: DEFF = 1 + (151–1) × 0.03 = 5.5. Although full compensation for the design effect would theoretically require a much larger sample, a pragmatic approach was adopted considering fieldwork constraints and the use of robust structural equation modeling techniques. Thus, the sample size was conservatively inflated by a factor of 1.5 to enhance representativeness and reduce sampling error while maintaining analytical precision. The final minimum target sample size was set at 680 participants (453 × 1.5). A total of 901 questionnaires were distributed, with 878 valid responses collected, yielding a response rate of 97.45%. Among these, 91 participants were from Beijing (planned minimum: 90), 549 from Sichuan (planned minimum: 227), and 238 from Yunnan (planned minimum: 136), all meeting or exceeding the planned sample sizes and satisfying regional representativeness requirements. All participants signed an electronic informed consent form prior to participation, fully understanding the study’ s purpose and content.

### 2.2 Methods and quality control

This study adopted a multi-center cross-sectional survey design, collecting data through convenience sampling. Data collection was coordinated by the Chinese Nursing Association. The director of the nursing department at the researcher’ s hospital distributed questionnaires to national committee members of the association, who then forwarded them to nursing department directors and head nurses in public hospitals in Beijing, Sichuan, and Yunnan, ultimately distributing them to registered nurses for completion. The questionnaires were administered electronically via the Wenjuanxing platform^1^ from December 26, 2024 to February 25, 2025, with nurses completing them anonymously online by scanning a QR code. To ensure completeness, all questions were set as mandatory, and each device was restricted to submitting only one response. To maintain data quality, based on pre-survey results (average response time of 362.24 s), questionnaires completed in <120 s were excluded. Additionally, collected data underwent logical consistency checks to eliminate responses with obvious contradictions. This study received approval from the Medical Research Ethics Committee of the researcher’ s institution [Ethics approval number: medical research 2024 ethics approval no. (202)]. Prior to participation, all respondents were required to review an electronic informed consent form on the questionnaire’ s homepage, which detailed the study’ s purpose, content, and privacy protection measures, and could proceed to complete the questionnaire only after providing consent. All data were anonymized to ensure the confidentiality and security of participants’ personal information and responses.

### 2.3 Survey instruments

#### 2.3.1 General information questionnaire

This study utilized a self-designed questionnaire to collect nurses’ general information, including gender, age group, ethnicity, marital status, educational level, professional title, years of work experience, work department, job content, whether they serve as a preceptor, AI-related training experience, province of the workplace, hospital type, hospital level, and whether the hospital is a teaching hospital. To further assess nurses’ intention to use AI, two additional questions were included: “Are you willing to use AI to assist in learning and work?”(USE) and “Are you willing to receive training on AI-related knowledge?”(LEA) These questions were scored using a 10-point Likert-type scale, where 0 represents “completely unwilling” and 10 represents “completely willing.” The average score of the two items was used to construct the “AI Usage Intention”(TODO as a custom code) variable for subsequent analysis. The internal consistency of this variable was high (Cronbach’s *α* = 0.924).

#### 2.3.2 General attitudes toward artificial intelligence scale (GAAIS)

The General Attitudes toward Artificial Intelligence Scale (GAAIS), developed by Schepman and Rodway (22), has been validated as an effective tool for measuring individuals’ positive and negative attitudes toward AI and has been successfully applied to assess attitudes among nurses (23). The scale comprises two subscales—positive attitudes and negative attitudes—with a total of 20 items, scored on a 5-point Likert-type scale. Items in the positive attitude subscale are scored directly (1 = strongly disagree, 5 = strongly agree), while items in the negative attitude subscale are reverse-scored (5 = strongly disagree, 1 = strongly agree). The total score ranges from 20 to 100, with higher scores indicating more positive attitudes. In this study, the Cronbach’ s *α* for the overall GAAIS was 0.903, with values of 0.952 and 0.955 for the positive and negative attitude subscales, respectively. Additionally, the scale’ s Kaiser-Meyer-Olkin (KMO) value was 0.945, and Bartlett’ s test of sphericity showed a significance level of <0.001, indicating excellent internal consistency and structural validity for the adapted Chinese version, making it suitable for this study.

#### 2.3.3 Artificial intelligence literacy scale (AILS)

The Artificial Intelligence Literacy Scale (AILS), developed by Wang et al. (24), is considered one of the ideal tools for assessing AI literacy due to its proposed four-dimensional structural model, encompassing awareness, usage, evaluation, and ethical capability. The scale aims to quantify core competencies of general users in the AI domain and has been validated and applied in groups such as students, teachers, and nurses (25–27). The AILS consists of four subscales—awareness, usage, evaluation, and ethics—with a total of 12 items, scored on a 7-point Likert-type scale (1 = strongly disagree, 7 = strongly agree). Three items are reverse-scored, and the total score ranges from 12 to 84, with higher scores indicating greater AI literacy. In this study, the overall Cronbach’s *α* for the AILS was 0.829, with a Kaiser-Meyer-Olkin (KMO) value of 0.894 and a Bartlett’s test of sphericity significance level <0.001, demonstrating good internal consistency and structural validity, making the scale suitable for this study.

#### 2.3.4 Artificial intelligence self-efficacy scale (AISES)

The Artificial Intelligence Self-Efficacy Scale (AISES), developed by Wang and Chuang (28), is designed to assess individuals’ perceived self-efficacy regarding specific features of AI technology. The scale has been widely applied and validated in groups such as students and educators (29). The AISES comprises 22 items, divided into four dimensions: Assistance, Anthropomorphic Interaction, Comfort with AI, and Technological Skills, scored on a 7-point Likert-type scale (1 = strongly disagree, 7 = strongly agree). The total score ranges from 22 to 154, with higher scores indicating stronger self-efficacy in using AI technologies/products. In this study, the overall Cronbach’ s *α* for the AISES was 0.980, with Cronbach’ s α values for the subscales (Assistance, Anthropomorphic Interaction, Comfort with AI, and Technological Skills) being 0.972, 0.937, 0.975, and 0.943, respectively. The scale’ s KMO value was 0.968, and Bartlett’ s test of sphericity showed a significance level <0.001, indicating excellent internal consistency and structural validity, making the scale suitable for this study.

### 2.4 Statistical analysis

This study utilized Epidata 3.1 for double data entry and logical checks to ensure data accuracy and consistency, with data analysis conducted using SPSS 26.0. To assess potential common method bias, Harman’ s single-factor test was applied to the General Attitudes toward Artificial Intelligence Scale (GAAIS), Artificial Intelligence Literacy Scale (AILS), and Artificial Intelligence Self-Efficacy Scale (AISES). Participants’ demographic characteristics and scale scores were presented through descriptive statistical analysis: demographic characteristics were expressed as frequencies and percentages (%), while continuous variables, such as scale scores, were assessed for normality. If normally distributed, they were described using mean ± standard deviation (x̄ ± s); otherwise, median and interquartile range [M (Q1, Q3)] were used. To correct for uneven regional sample distribution, case weighting adjustments were applied based on the total number of nurses in Beijing (126,111), Sichuan (318,267), and Yunnan (189,852) as reported in the China Statistical Yearbook 2023, and the actual sample sizes (91, 549, and 238, respectively). The weighting variable was entered into AMOS using the “sample weight” function and applied in the subsequent structural equation modeling analysis. The weight data were processed in SPSS, and AMOS successfully implemented the weighting.

For group comparisons of continuous variables, appropriate methods were selected based on normality and homogeneity of variance tests: for two-group comparisons, independent samples *t*-tests were used if normality and homogeneity of variance were satisfied; otherwise, Mann–Whitney U tests were employed. For comparisons involving three or more groups, one-way analysis of variance (ANOVA) with *post-hoc* multiple comparisons was used if normality and homogeneity of variance were met; otherwise, non-parametric tests were applied. Pearson correlation analysis was used to evaluate the relationships among GAAIS, AILS, and AISES scores, with correlation coefficients ranging from −1 to +1; values closer to 1 indicate stronger correlations, and 0 indicates no correlation. Structural equation modeling (SEM) was performed using AMOS 26.0 to analyze the path relationships among general attitudes toward AI, AI literacy, AI self-efficacy, and usage intention. In this study, we decided to use composite scores to simplify the analysis process. We recognize that using composite scores may introduce some bias, as it may obscure the independent contributions of each item. To minimize this bias, we ensured that the three scales used are well-established and validated, with strong internal consistency. While item-level modeling could theoretically provide more detailed analysis, the complexity of the models could lead to overly complicated results, especially when there are many items in each scale, and the items are highly correlated. This approach is consistent with prior research and widely accepted in the field. All statistical tests adopted a two-tailed significance level of *α* = 0.05.

## 3 Results

### 3.1 General characteristics of participants

This study distributed 901 questionnaires, of which 2 respondents declined participation, and 21 questionnaires were excluded due to completion times of <120 s. Ultimately, 878 valid questionnaires were collected, yielding an effective response rate of 97.45%. Among the respondents, females accounted for 94.08% (*n =* 826), those aged 40 and below comprised 84.03% (*n =* 738), Han ethnicity represented 92.71% (*n =* 814), and married individuals made up 70.62% (*n =* 620). The majority had a bachelor’ s degree or undergraduate education (75.74%, *n =* 665), and those with junior or intermediate professional titles accounted for 82.92% (*n =* 728). Years of work experience were relatively evenly distributed. The predominant work department was pediatrics (49.32%, *n =* 433), with 92.37% (*n =* 811) engaged in clinical nursing. Preceptors comprised 56.04% (*n =* 492), and only 14.24% (*n =* 125) had received professional AI-related training. Regarding workplace, specialty hospitals and general hospitals accounted for 49.66% (*n =* 436) and 50.34% (*n =* 442), respectively; 57.52% (*n =* 505) of respondents were from Grade IIIA hospitals, and 71.53% (*n =* 628) worked in teaching hospitals. The mean score of the “AI Usage Intention” variable was 8.40 ± 1.92, indicating a generally high level of willingness to use AI among participants. Detailed information is presented in Table 1.

**Table 1:** Differences in AI general attitudes, AI literacy, and AI self-efficacy across general characteristics of participants (*N* = 878).

Variable	Category	Frequency	Percentage (100%)	Total score of AI general attitudes			Total score of AI literacy			Total score of AI self-efficacy		
				*M* ± *SD*	*t/F*	*P*	*M* ± *SD*	*t/F*	*P*	*M* ± *SD*	*t/F*	*P*
Gender	Female	826	94.08%	69.38 ± 10.34	0.587	0.557	56.37 ± 8.68	1.411	0.158	107.90 ± 22.32	−0.102	0.919
	Male	52	5.92%	68.52 ± 9.81			54.63 ± 7.11			108.23 ± 23.00		
Age group (years)	≤30	342	38.59%	68.78 ± 9.83	2.095	0.099	56.20 ± 8.17	1.033	0.377	107.38 ± 21.69	0.809	0.489
	31⁓40	399	45.44%	69.15 ± 10.38			56.39 ± 9.08			107.49 ± 22.47		
	41⁓50	121	13.78%	71.06 ± 11.41			56.55 ± 8.40			110.26 ± 24.22		
	≥51	16	1.82%	72.75 ± 8.25			52.63 ± 6.42			112.75 ± 18.30		
Ethnicity	Han	814	92.71%	69.37 ± 10.34	0.332	0.740	56.28 ± 8.74	0.267*	0.790	107.69 ± 22.43	−1.126	0.240
	Other	64	7.29%	68.92 ± 9.89			56.05 ± 6.67			110.95 ± 21.17		
Marital status	Unmarried	235	26.77%	69.00 ± 9.30	0.510*	0.603	56.69 ± 8.10	0.661*	0.520	108.43 ± 21.50	0.489	0.613
	Married	620	70.62%	69.36 ± 10.50			56.06 ± 8.72			107.59 ± 22.53		
	Divorced	23	2.62%	71.91 ± 14.24			57.57 ± 10.37			111.87 ± 26.29		
Educational level	Associate degree or below	180	20.5%	67.87 ± 10.33	6.668	0.001	54.91 ± 7.93	18.221	<0.001	107.17 ± 24.38	5.764*	0.005
	Bachelor’s degree or undergraduate	665	75.74%	69.46 ± 10.30			56.22 ± 8.54			107.54 ± 21.72		
	Master’s degree or above	33	3.76%	74.85 ± 8.27			64.55 ± 9.03			119.85 ± 20.42		
Professional title	None	91	10.36%	68.41 ± 9.32	2.386	0.068	56.25 ± 8.07	0.846	0.469	109.25 ± 22.30	1.997	0.116
	Junior	379	43.17%	69.04 ± 10.58			56.30 ± 8.68			107.58 ± 22.79		
	Intermediate	349	39.75%	69.33 ± 10.35			55.96 ± 8.69			106.88 ± 21.94		
	Senior	59	6.72%	72.64 ± 9.27			57.88 ± 8.35			114.25 ± 21.28		
Years of Work experience (years)	≤5	217	24.72%	68.35 ± 8.98	2.555*	0.055	56.35 ± 7.90	0.071	0.975	107.88 ± 21.63	0.389	0.761
	6⁓10	233	26.54%	70.06 ± 10.96			56.38 ± 8.81			107.35 ± 22.04		
	11⁓15	233	26.54%	68.47 ± 9.90			56.05 ± 8.90			107.31 ± 22.42		
	≥16	195	22.21%	70.59 ± 11.19			56.30 ± 8.80			109.39 ± 23.48		
Work department	Adult general	109	12.41%	67.99 ± 9.55	3.878*	0.010	55.53 ± 8.38	3.946*	0.009	109.35 ± 25.16	3.393*	0.018
	Obstetrics and gynecology	207	23.58%	68.79 ± 9.82			55.37 ± 8.14			106.19 ± 20.9		
	Pediatrics	433	49.32%	70.44 ± 10.98			57.25 ± 9.08			109.68 ± 22.76		
	Non-inpatient wards	129	14.69%	67.62 ± 8.89			55.01 ± 7.48			103.6 ± 20.02		
Job content	Clinical nursing	811	92.37%	69.18 ± 10.39	−1.489	0.137	56.14 ± 8.55	−1.511	0.131	107.64 ± 22.39	−1.287	0.199
	Administration/teaching/research/other	67	7.63%	71.13 ± 9.16			57.79 ± 9.09			111.30 ± 21.78		
Preceptor	Yes	492	56.04%	70.06 ± 10.53	2.367	0.018	56.90 ± 8.67	2.473	0.014	109.58 ± 22.31	2.486	0.013
	No	386	43.96%	68.41 ± 9.95			55.46 ± 8.45			105.81 ± 22.25		
AI training experience	No training received whatsoever	387	44.08%	69.35 ± 10.48	0.119	0.888	55.32 ± 8.51	5.204	0.006	104.67 ± 21.69	11.763*	<0.001
	No professional training but received related thematic training or self-study	366	41.69%	69.46 ± 10.27			56.70 ± 8.35			108.54 ± 21.68		
	Received professional training	125	14.24%	68.94 ± 9.94			57.94 ± 9.27			116.18 ± 24.11		
Province of workplace	Sichuan	549	62.53%	69.50 ± 9.84	8.190*	<0.001	56.55 ± 8.37	9.816*	<0.001	107.44 ± 21.16	6.554*	0.002
	Yunnan	238	27.11%	67.46 ± 9.53			54.49 ± 8.17			105.83 ± 23.64		
	Beijing	91	10.36%	73.21 ± 13.46			59.23 ± 10.02			116.33 ± 24.17		
Hospital type	Specialty hospital	436	49.66%	70.38 ± 10.23	3.010	0.003	57.16 ± 8.66	3.066	0.002	109.31 ± 21.45	1.835*	0.067
	General hospital	442	50.34%	68.30 ± 10.28			55.39 ± 8.46			106.55 ± 23.14		
Hospital level	Grade IIIA	505	57.52%	71.10 ± 10.55	11.417*	0.001	57.70 ± 8.70	11.957	<0.001	111.22 ± 21.89	9.452	<0.001
	Grade IIB	136	15.49%	67.16 ± 8.92			54.09 ± 8.06			103.08 ± 21.65		
	Grade IIC	2	0.23%	68.50 ± 0.71			61.00 ± 7.07			124.00 ± 42.43		
	Grade II or below	235	26.77%	66.80 ± 9.81			54.40 ± 8.10			103.51 ± 22.43		
Teaching hospital	Yes	628	71.53%	69.85 ± 10.62	2.500*	0.018	56.78 ± 8.69	2.790	0.005	108.96 ± 22.43	2.176	0.030
	No	250	28.47%	68.03 ± 9.36			54.99 ± 8.26			105.33 ± 21.96		
Willingness to Use artificial intelligence to assist in learning and work		8.40 ± 1.93										
Willingness to receive training on AI-related knowledge		8.40 ± 1.91										

*Indicates heterogeneity of variance, Welch’s *t-*test was used.

### 3.2 Scores and influencing factors of AI general attitudes, AI literacy, and AI self-efficacy

The mean scores for the General Attitudes toward Artificial Intelligence Scale (GAAIS), Artificial Intelligence Literacy Scale (AILS), and Artificial Intelligence Self-Efficacy Scale (AISES) were 69.33 ± 10.31, 56.27 ± 8.60, and 107.92 ± 22.35, respectively. Analysis of demographic characteristics revealed significant differences in scale scores across different groups (Table 1).

AI general attitude scores: significant differences were observed based on educational level (*p* = 0.001), work department (*p* = 0.010), preceptor status (*p* = 0.018), province of workplace (*p* < 0.001), hospital type (*p* = 0.003), hospital level (*p* = 0.001), and teaching hospital status (*p* = 0.018).

AI literacy scores: significant differences were found based on educational level (*p* < 0.001), work department (*p* = 0.009), preceptor status (*p* = 0.014), AI-related training experience (*p* = 0.006), province of workplace (*p* < 0.001), hospital type (*p* = 0.002), hospital level (*p* < 0.001), and teaching hospital status (*p* = 0.005).

AI self-efficacy scores: significant differences were identified based on educational level (*p* = 0.005), work department (*p* = 0.018), preceptor status (*p* = 0.013), AI-related training experience (*p* < 0.001), province of workplace (*p* = 0.002), hospital level (*p* < 0.001), and teaching hospital status (*p* = 0.005).

### 3.3 Common method Bias test

This study employed Harman’ s single-factor test to assess common method bias. The results showed five factors with eigenvalues >1, with the first factor accounting for 20.62% of the variance and the cumulative variance of the top five factors explaining 73.53%. Since the variance explained by the first factor was well below the critical threshold of 50%, it indicates that no single factor accounted for the majority of the variance, suggesting that common method bias was not significant in this study.

### 3.4 Group differences analysis of AI general attitudes, AI literacy, and AI self-efficacy

T-tests, Mann–Whitney U tests, or one-way analysis of variance (ANOVA) were used to examine differences in scores of the General Attitudes toward Artificial Intelligence Scale (GAAIS), Artificial Intelligence Literacy Scale (AILS), and Artificial Intelligence Self-Efficacy Scale (AISES) across various demographic characteristics (Table 1). The results indicated that preceptors had significantly higher total scores for GAAIS, AILS, and AISES compared to non-preceptors (*p* < 0.05), and nurses in teaching hospitals scored significantly higher than those in non-teaching hospitals (*p* < 0.05). By hospital type, nurses in specialty hospitals had higher GAAIS and AILS total scores than those in general hospitals (*p* < 0.05), but no statistically significant difference was found in AISES scores (*p* > 0.05).

For data with equal variances, the LSD method is used for *post-hoc* multiple comparisons, and Cohen’s d is supplemented to measure the effect size. For data with unequal sample sizes and unequal variances, the Games-Howell method is used for *post-hoc* multiple comparisons, and Glass’ Delta based on the control group’ s standard deviation is supplemented to calculate the effect size. All statistical tests adopted a two-tailed significance level of *α* = 0.05. The results of multiple comparisons are detailed in Table 2.

1. Educational level: the GAAIS, AILS, and AISES scores of the master’ s degree or above group were significantly higher than those of the associate degree or below group and the bachelor’ s degree or undergraduate group (*p* < 0.05). No statistically significant differences were observed between the associate degree or below group and the bachelor’s degree or undergraduate group (*p* > 0.05).
2. Work department: significant differences were found in GAAIS, AILS, and AISES scores between pediatric nurses and nurses in non-inpatient wards (*p* < 0.05). Pairwise comparisons among other departments showed no statistically significant differences (*p* > 0.05).
3. Province of workplace: after case weighting adjustments based on the total number of nurses and actual sample sizes in Beijing, Sichuan, and Yunnan, Beijing nurses’ GAAIS, AILS, and AISES scores were significantly higher than those of Sichuan nurses (*p* < 0.05), and Sichuan nurses’ scores were significantly higher than those of Yunnan nurses (*p* < 0.05).
4. Hospital level: nurses in Grade IIIA hospitals had significantly higher GAAIS, AILS, and AISES scores than nurses in other hospital levels (*p* < 0.05). No statistically significant differences were found among nurses in other hospital levels (*p* > 0.05).
5. AI-related training experience: nurses who received AI-related training (including self-study or professional training) had significantly higher AILS scores than those without any training (*p* < 0.05), but no significant differences were found between the self-study and professional training groups (*p* > 0.05). For AISES scores, nurses who received professional training scored significantly higher than those with self-study or only related thematic training (*p* < 0.05), and the latter group scored significantly higher than those without any training (*p* < 0.05).

**Table 2:** Multiple comparisons of AI general attitudes, AI literacy, and AI self-efficacy across general characteristics of participants (*N* = 878).

Variable	Dependent variable	Multiple comparison test method	(I) Group	(J) Group	MD (I-J)	SE	*P*	95% CI LowerBound	95% CI UpperBound	Cohen’s d /Glass’ delta	95%CI LowerBound	95%CI UpperBound
Educational level	AI general attitudes	LSD	Associate degree or below	Bachelor’s degree or undergraduate	−1.583	0.860	0.066	−3.272	0.105	−0.154	−0.318	0.011
				Master’s degree or above	−6.976*	1.939	<0.001	−10.782	−3.171	−0.695	−1.071	−0.317
			Bachelor’s degree or undergraduate	Associate degree or below	1.583	0.860	0.066	−0.105	3.272	0.154	−0.011	0.318
				Master’s degree or above	−5.393*	1.826	0.003	−8.977	−1.809	−0.528	−0.878	−0.177
			Master’s degree or above	Associate degree or below	6.976*	1.939	<0.001	3.171	10.782	0.695	0.317	1.071
				Bachelor’s degree or undergraduate	5.393*	1.826	0.003	1.809	8.977	0.528	0.177	0.878
	AI literacy	LSD	Associate degree or below	Bachelor’s degree or undergraduate	−1.311	0.709	0.065	−2.703	0.080	−0.156	−0.321	0.009
				Master’s degree or above	−9.634*	1.598	<0.001	−12.770	−6.499	−1.189	−1.576	−0.799
			Bachelor’s degree or undergraduate	Associate degree or below	1.311	0.709	0.065	−0.080	2.703	0.156	−0.009	0.321
				Master’s degree or above	−8.323*	1.505	<0.001	−11.276	−5.370	−0.972	−1.325	−0.618
			Master’s degree or above	Associate degree or below	9.634*	1.598	<0.001	6.499	12.770	1.189	0.799	1.576
				Bachelor’s degree or undergraduate	8.323*	1.505	<0.001	5.370	11.276	0.972	0.618	1.325
	AI self-efficacy	Games-Howell	Associate degree or below	Bachelor’s degree or undergraduate	−0.170	0.074	0.055	−0.343	0.003	−0.017	−0.182	0.148
				Master’s degree or above	−13.903*	0.153	<0.001	−14.261	−13.545	−0.621	−1.018	−0.216
			Bachelor’s degree or undergraduate	Associate degree or below	0.170	0.074	0.055	−0.003	0.343	0.015	−0.150	0.180
				Master’s degree or above	−13.733*	0.141	<0.001	−14.064	−13.401	−0.603	−0.978	−0.220
			Master’s degree or above	Associate degree or below	13.903*	0.153	<0.001	13.545	14.261	0.520	0.144	0.894
				Bachelor’s degree or undergraduate	13.733*	0.141	<0.001	13.401	14.064	0.567	0.216	0.917
Work department	AI general attitudes	Games-Howell	Adult general	Obstetrics and gynecology	−0.801	1.141	0.896	−3.755	2.152	−0.082	−0.314	0.151
				Pediatrics	−2.450	1.056	0.097	−5.187	0.287	−0.223	−0.433	−0.012
				Non-inpatient wards	0.371	1.204	0.990	−2.745	3.486	0.042	−0.213	0.297
			Obstetrics and gynecology	Adult general	0.801	1.141	0.896	−2.152	3.755	0.084	−0.148	0.316
				Pediatrics	−1.649	0.863	0.225	−3.874	0.576	−0.150	−0.316	0.016
				Non-inpatient wards	1.172	1.039	0.672	−1.512	3.856	0.132	−0.089	0.352
			Pediatrics	Adult general	2.450	1.056	0.097	−0.287	5.187	0.257	0.043	0.469
				Obstetrics and gynecology	1.649	0.863	0.225	−0.576	3.874	0.168	0.001	0.334
				Non-inpatient wards	2.821*	0.944	0.016	0.379	5.263	0.317	0.116	0.517
			Non-inpatient wards	Adult general	−0.371	1.204	0.990	−3.486	2.745	−0.039	−0.294	0.216
				Obstetrics and gynecology	−1.172	1.039	0.672	−3.856	1.512	−0.119	−0.339	0.101
				Pediatrics	−2.821*	0.944	0.016	−5.263	−0.379	−0.257	−0.454	−0.059
	AI literacy	Games-Howell	Adult general	Obstetrics and gynecology	0.160	0.982	0.998	−2.382	2.703	0.020	−0.212	0.252
				Pediatrics	−1.722	0.913	0.238	−4.091	0.647	−0.190	−0.400	0.021
				Non-inpatient wards	0.524	1.038	0.958	−2.163	3.212	0.070	−0.185	0.325
			Obstetrics and gynecology	Adult general	−0.160	0.982	0.998	−2.703	2.382	−0.019	−0.251	0.213
				Pediatrics	−1.882	0.715	0.051	−3.770	0.006	−0.207	−0.373	−0.041
				Non-inpatient wards	0.364	0.868	0.975	−1.879	2.608	0.049	−0.171	0.269
			Pediatrics	Adult general	1.722	0.913	0.238	−0.647	4.091	0.206	−0.007	0.417
				Obstetrics and gynecology	1.882	0.715	0.051	−0.006	3.770	0.231	0.064	0.398
				Non-inpatient wards	2.246*	0.790	0.025	0.203	4.290	0.300	0.100	0.500
			Non-inpatient wards	Adult general	−0.524	1.038	0.958	−3.212	2.163	−0.063	−0.318	0.193
				Obstetrics and gynecology	−0.364	0.868	0.975	−2.608	1.879	−0.045	−0.265	0.175
				Pediatrics	−2.246*	0.790	0.025	−4.290	−0.203	−0.287	−0.445	−0.050
	AI self-efficacy	Games-Howell	Adult general	Obstetrics and gynecology	3.160	2.814	0.676	−4.133	10.454	0.151	−0.081	0.383
				Pediatrics	−0.333	2.646	0.999	−7.205	6.539	−0.015	−0.225	0.195
				Non-inpatient wards	5.744	2.986	0.221	−1.989	13.477	0.187	0.029	0.344
			Obstetrics and gynecology	Adult general	−3.160	2.814	0.676	−10.454	4.133	−0.126	−0.358	0.107
				Pediatrics	−3.493	1.818	0.221	−8.182	1.197	−0.153	−0.319	0.013
				Non-inpatient wards	2.584	2.284	0.671	−3.320	8.488	0.129	−0.092	0.349
			Pediatrics	Adult general	0.333	2.646	0.999	−6.539	7.205	0.013	−0.197	0.223
				Obstetrics and gynecology	3.493	1.818	0.221	−1.197	8.182	0.167	0.001	0.333
				Non-inpatient wards	6.077*	2.075	0.019	0.709	11.445	0.303	0.103	0.503
			Non-inpatient wards	Adult general	−5.744	2.986	0.221	−13.477	1.989	−0.208	−0.385	0.029
				Obstetrics and gynecology	−2.584	2.284	0.671	−8.488	3.320	−0.124	−0.344	0.097
				Pediatrics	−6.077*	2.075	0.019	−11.445	−0.709	−0.267	−0.464	−0.069
AI training experience	AI general attitudes	LSD	No training received whatsoever	Received related thematic training or self-study	−0.110	0.752	0.884	−1.586	1.366	−0.011	−0.154	0.132
				Received professional training	0.410	1.061	0.699	−1.673	2.493	0.040	−0.162	0.241
			Received related thematic training or self-study	No training received whatsoever	0.110	0.752	0.884	−1.366	1.586	0.011	−0.132	0.154
				Received Professional Training	0.520	1.069	0.626	−1.577	2.618	0.051	−0.152	0.254
			Received professional training	No training received whatsoever	−0.410	1.061	0.699	−2.493	1.673	−0.040	−0.241	0.162
				Received related thematic training or self-study	−0.520	1.069	0.626	−2.618	1.577	−0.051	−0.254	0.152
	AI literacy	LSD	No training received whatsoever	Received related thematic training or self-study	−1.376*	0.624	0.028	−2.601	−0.151	−0.363	−0.406	−0.220
				Received professional training	−2.616*	0.881	0.003	−4.344	−0.887	−0.301	−0.503	−0.098
			Received related thematic training or self-study	No training received whatsoever	1.376*	0.624	0.028	0.151	2.601	0.363	0.220	0.406
				Received professional training	−1.239	0.887	0.163	−2.980	0.501	−0.144	−0.347	0.059
			Received professional training	No training received whatsoever	2.616*	0.881	0.003	0.887	4.344	0.301	0.098	0.503
				Received related thematic training or self-study	1.239	0.887	0.163	−0.501	2.980	0.144	−0.059	0.347
	AI self-efficacy	Games-Howell	No training received whatsoever	Received related thematic training or self-study	−3.869*	1.581	0.039	−7.582	−0.156	−0.378	−0.522	−0.135
				Received professional training	−11.512*	2.422	<0.001	−17.233	−5.792	−0.477	−0.687	−0.266
			Received related thematic training or self-study	No training received whatsoever	3.869*	1.581	0.039	0.156	7.582	0.378	0.135	0.522
				Received Professional Training	−7.643*	2.436	0.006	−13.396	−1.890	−0.317	−0.523	−0.110
			Received professional training	No training received whatsoever	11.512*	2.422	<0.001	5.792	17.233	0.531	0.325	0.736
				Received related thematic training or self-study	7.643*	2.436	0.006	1.890	13.396	0.353	0.148	0.557
Province of workplace (after case weighting)	AI general Attitudes	Games-Howell	Sichuan	Yunnan	2.041*	0.028	<0.001	1.975	2.106	0.315	0.209	0.420
				Beijing	−3.706*	0.042	<0.001	−3.803	−3.609	−0.377	−0.383	−0.370
			Yunnan	Sichuan	−2.041*	0.028	<0.001	−2.106	−1.975	−0.307	−0.413	−0.202
				Beijing	−5.747*	0.044	<0.001	−5.849	−5.644	−0.559	−0.597	−0.522
			Beijing	Sichuan	3.706*	0.042	<0.001	3.609	3.803	0.377	0.370	0.383
				Yunnan	5.747*	0.044	<0.001	5.644	5.849	0.605	0.597	0.612
	AI literacy	Games-Howell	Sichuan	Yunnan	2.059*	0.024	<0.001	2.003	2.115	0.353	0.247	0.458
				Beijing	−2.684*	0.032	<0.001	−2.759	−2.610	−0.299	−0.376	−0.263
			Yunnan	Sichuan	−2.059*	0.024	<0.001	−2.115	−2.003	−0.346	−0.452	−0.240
				Beijing	−4.743*	0.034	<0.001	−4.822	−4.664	−0.576	−0.583	−0.569
			Beijing	Sichuan	2.684*	0.032	<0.001	2.610	2.759	0.321	0.314	0.327
				Yunnan	4.743*	0.034	<0.001	4.664	4.822	0.582	0.574	0.589
	AI self-efficacy	Games-Howell	Sichuan	Yunnan	1.605*	0.066	<0.001	1.451	1.759	0.368	0.162	0.474
				Beijing	−8.893*	0.077	<0.001	−9.074	−8.711	−0.390	−0.477	−0.363
			Yunnan	Sichuan	−1.605*	0.066	<0.001	−1.759	−1.451	−0.376	−0.482	−0.170
				Beijing	−10.498*	0.087	<0.001	−10.701	−10.295	−0.437	−0.444	−0.429
			Beijing	Sichuan	8.893*	0.077	<0.001	8.711	9.074	0.421	0.414	0.427
				Yunnan	10.498*	0.087	<0.001	10.295	10.701	0.445	0.438	0.452
Hospital level^b^	AI general attitudes	Games-Howell	Grade IIIA	Grade IIB	3.941*	0.898	<0.001	1.824	6.058	0.442	0.244	0.638
				Grade II or below	4.307*	0.794	<0.001	2.441	6.173	0.439	0.279	0.598
			Grade IIB	Grade IIIA	−3.941*	0.898	<0.001	−6.058	−1.824	−0.385	−0.576	−0.195
				Grade II or below	0.366	0.997	0.928	−1.983	2.715	0.037	−0.174	0.248
			Grade II or Below	Grade IIIA	−4.307*	0.794	<0.001	−6.173	−2.441	−0.408	−0.565	−0.251
				Grade IIB	−0.366	0.997	0.928	−2.715	1.983	−0.041	−0.252	0.170
	AI literacy	LSD	Grade IIIA	Grade IIB	3.617*	0.816	<0.001	2.015	5.218	0.422	0.231	0.613
				Grade II or below	3.309*	0.667	<0.001	2.000	4.618	0.389	0.232	0.545
			Grade IIB	Grade IIIA	−3.617*	0.816	<0.001	−5.218	−2.015	−0.422	−0.613	−0.231
				Grade II or below	−0.308	0.910	0.735	−2.093	1.478	−0.038	−0.249	0.173
			Grade II or below	Grade IIIA	−3.309*	0.667	<0.001	−4.618	−2.000	−0.389	−0.545	−0.232
				Grade IIB	0.308	0.910	0.735	−1.478	2.093	0.038	−0.173	0.249
	AI self-efficacy	LSD	Grade IIIA	Grade IIB	8.137*	2.125	<0.001	3.966	12.308	0.373	0.182	0.563
				Grade II or below	7.707*	1.737	<0.001	4.298	11.116	0.349	0.193	0.505
			Grade IIB	Grade IIIA	−8.137*	2.125	<0.001	−12.308	−3.966	−0.373	−0.563	−0.182
				Grade II or below	−0.430	2.370	0.856	−5.081	4.222	−0.019	−0.231	0.192
			Grade II or below	Grade IIIA	−7.707*	1.737	<0.001	−11.116	−4.298	−0.349	−0.505	−0.193
				Grade IIB	0.430	2.370	0.856	−4.222	5.081	0.019	−0.192	0.231

*Significance level of mean difference is 0.05. ^b^Indicates that the sample size within the group is <3, lacking group representativeness, and thus *post-hoc* testing was not conducted for this group.

### 3.5 Correlation analysis of AI general attitudes, AI literacy, AI self-efficacy, and usage intention

After applying case weighting adjustments for the province of the workplace, partial correlation analysis was conducted to control for the effects of variables including educational level, work department, preceptor status, AI-related training experience, province of workplace, hospital type, hospital level, and teaching hospital status. This analysis examined the correlations among the total scores of the General Attitudes toward Artificial Intelligence Scale (GAAIS), Artificial Intelligence Literacy Scale (AILS), Artificial Intelligence Self-Efficacy Scale (AISES), and AI usage intention (Table 3). The results showed significant positive correlations between GAAIS total scores and AILS total scores (r = 0.549, *p* < 0.001) and AISES total scores (r = 0.567, *p* < 0.001). AILS total scores were also significantly positively correlated with AISES total scores (r = 0.684, *p* < 0.001). Additionally, AI usage intention exhibited significant positive correlations with the total scores of GAAIS, AILS, and AISES (*p* < 0.001).

**Table 3:** Correlation analysis of AI general attitudes, AI literacy, AI self-efficacy, and usage intention (*n* = 878).

Dimension	M ± SD	TODO-USE	TODO-LEA	GAAIS	AILS	AISES
TODO-USE (willingness to use AI to assist in learning?)	8.43 ± 1.96	1				
TODO-LEA (willingness to receive AI-related training?)	8.41 ± 1.92	0.837***	1			
GAAIS (total score of general attitudes toward AI)	69.63 ± 10.73	0.409***	0.403***	1		
AILS (total score of AI literacy)	56.46 ± 8.80	0.372***	0.378 ***	0.549***	1	
AISES (total score of AI self-efficacy)	108.72 ± 22.81	0.429***	0.379***	0.567***	0.684***	1

***P < 0.001 (two-tailed).

### 3.6 Mediating effect analysis of AI general attitudes, AI literacy, AI self-efficacy, and usage intention

**Figure 2:**
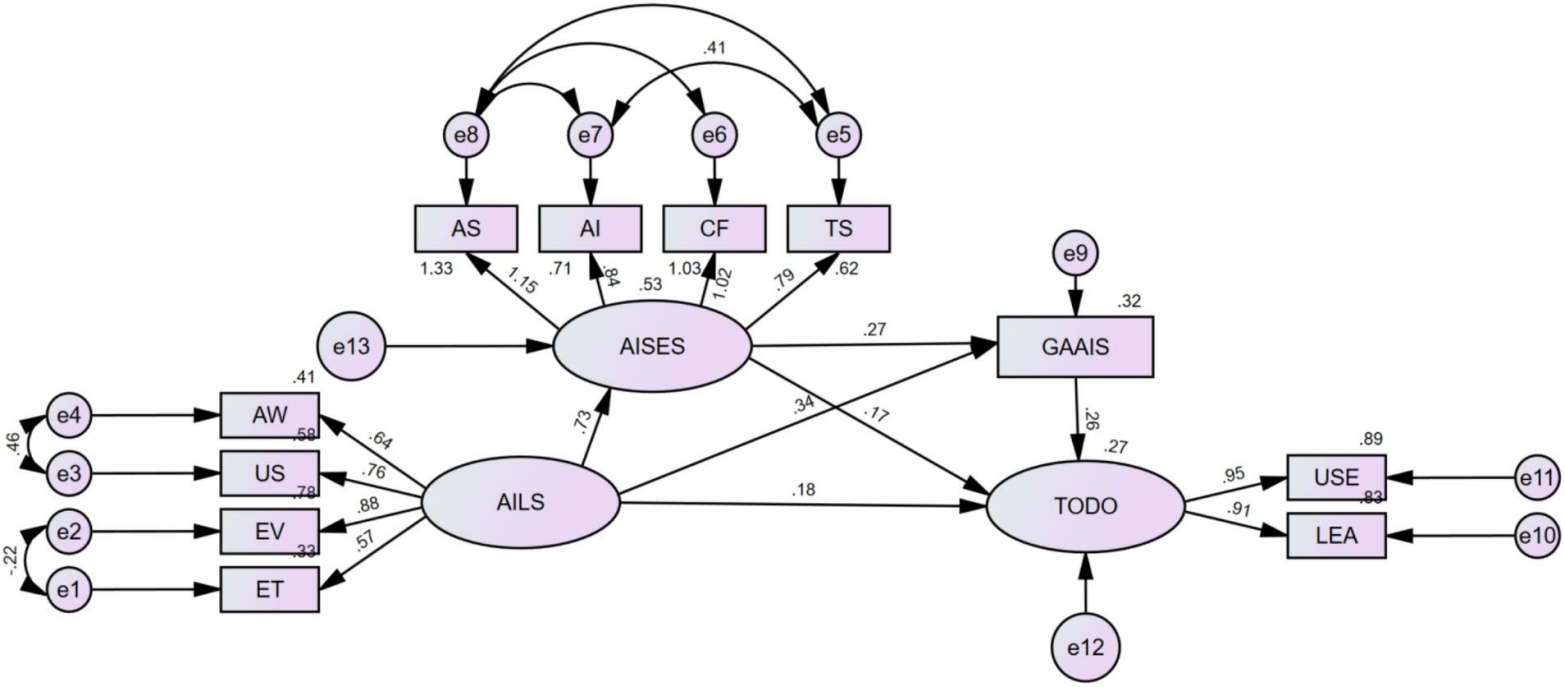
Mediation effect analysis of AI general attitudes, AI literacy, AI self-efficacy, and usage intention.

Using AI usage intention (TODO) as the dependent variable, AI Literacy Scale (AILS) scores as the independent variable, and AI Self-Efficacy Scale (AISES) scores and General Attitudes toward Artificial Intelligence Scale (GAAIS) scores as mediating variables, a structural equation model (SEM) was constructed using AMOS 26.0 to analyze mediating effects. The model fit results were as follows: *χ*^2^/df = 4.581, RMSEA = 0.0824, TLI = 0.901, CFI = 0.940, SRMR < 0.049. Compared to the fit criteria (*χ*^2^/df < 5, RMSEA < 0.08, TLI and CFI > 0.90, SRMR < 0.08), the *χ*^2^/df and RMSEA values are slightly above the ideal threshold, possibly due to the large sample size (*n =* 878). However, CFI, TLI, and SRMR met the criteria for good fit, indicating that the overall model fit was adequate (Figure 2).

The path coefficient analysis of the SEM revealed the following (Table 4): the path coefficient from AILS to AISES was 0.728, from AILS to GAAIS was 0.343, and from AILS to TODO was 0.180; the path coefficient from AISES to GAAIS was 0.274 and from AISES to TODO was 0.172; the path coefficient from GAAIS to TODO was 0.261. All path coefficients were statistically significant (*p* < 0.001), indicating significant direct effects among the variables.

**Table 4:** Mediation effect analysis of AI general attitudes, AI literacy, AI self-efficacy, and usage intention (*n* = 878).

Parameter	Estimate	S. E.	C. R.	*P*	Label
AISES ← AILS	0.728	0.073	13.639	<0.001	a
GAAIS ← AISES	0.274	0.047	7.237	<0.001	b
GAAIS ← AILS	0.343	0.086	6.852	<0.001	d2
TODO ← AILS	0.180	0.084	3.523	<0.001	x
TODO ← AISES	0.172	0.043	4.881	<0.001	d1
TODO ← GAAIS	0.261	0.035	6.927	<0.001	c

The Bootstrap method (5,000 resamples) was used to test the mediation effects, with results as follows (Table 5):

1. Path 1: AILS → AISES → TODO: The mediation effect value was 0.207.
2. (95% CI: 0.125–0.327, *p* < 0.001), accounting for 21.41% of the total effect.
3. Path 2: AILS → AISES → GAAIS → TODO: The mediation effect value was 0.082 (95% CI: 0.046–0.145, *p* < 0.001), accounting for 8.48% of the total effect.
4. Path 3: AILS → GAAIS → TODO: The mediation effect value was 0.141 (95% CI: 0.090–0.215, *p* < 0.001), accounting for 14.58% of the total effect.
5. Direct effect: AILS → TODO: The effect value was 0.295 (95% CI: 0.148–0.442, *p* = 0.002), accounting for 30.51% of the total effect.
6. Direct effect: GAAIS → TODO: The effect value was 0.242 (95% CI: 0.187–0.315, *p* < 0.001), accounting for 25.03% of the total effect.
7. Total effect: the total effect value was 0.967 (95% CI: 0.828–1.143, *p* < 0.001).

**Table 5:** Decomposition table of mediation effects for AI general attitudes, AI literacy, AI self-efficacy, and usage intention (*n* = 878).

Parameter	Estimate	95% CI		*P*	Proportion
		Lower	Upper		
TODO ← AISES ← AILS	0.207	0.125	0.327	<0.001	21.41%
TODO ← GAAIS ← AISES ← AILS	0.082	0.046	0.145	<0.001	8.48%
TODO ← GAAIS ← AILS	0.141	0.090	0.215	<0.001	14.58%
TODO ← AILS	0.295	0.148	0.442	0.002	30.51%
TODO ← GAAIS	0.242	0.187	0.315	<0.001	25.02%
Total	0.967	0.828	1.143	<0.001	

The 95% confidence intervals for all mediation effects did not include 0, indicating that the mediation effects were significant. The influence pathways of AI literacy on usage intention through AI self-efficacy and general AI attitudes were both confirmed, with the direct effect (30.51%) and the mediation effect through AI self-efficacy (21.41%) accounting for the largest proportions.

## 4 Discussion

### 4.1 Influence of demographic characteristics on AI general attitudes, AI literacy, and AI self-efficacy

#### 4.1.1 Influence of hospital type and level

This study found that nurses in specialty hospitals had significantly higher scores on the General Attitudes toward Artificial Intelligence Scale (GAAIS) and the Artificial Intelligence Literacy Scale (AILS) compared to those in general hospitals (*p* < 0.05), but no significant differences were observed in Artificial Intelligence Self-Efficacy Scale (AISES) scores. Nurses in Grade IIIA hospitals scored significantly higher on GAAIS, AILS, and AISES than those in other hospital levels (*p* < 0.05), with no significant differences among other hospital levels.

The higher GAAIS and AILS scores among specialty hospital nurses may be attributed to their frequent use of specific AI tools (e.g., clinical decision support systems or imaging analysis), which increases familiarity and positive attitudes toward AI. However, the lack of significant differences in AISES scores suggests that self-efficacy may depend more on individual training than hospital environment. Lambert et al. (30) indicated that the frequency of AI tool usage in hospitals significantly influences healthcare professionals’ technology acceptance, but confidence enhancement requires training. General hospitals, due to their broad service scope, may rely less on specific AI tools, resulting in lower scores. The highest scores among Grade IIIA hospital nurses reflect their technological resources and training opportunities. As top-tier medical institutions in China, Grade IIIA hospitals often lead in adopting AI technologies (e.g., intelligent monitoring systems) and provide more learning opportunities, enhancing nurses’ literacy, attitudes, and confidence. Maleki Varnosfaderani et al. (31) noted that advanced hospitals, with robust technological infrastructure, are more likely to promote AI application in clinical settings. Other hospital levels, limited by resources, show less AI integration and no significant score differences. This suggests that specialty hospitals should strengthen AI applications to improve attitudes and literacy, general hospitals need increased exposure to technology, and Grade IIIA hospitals can support lower-level hospitals through training and technological assistance to bridge the gap.

#### 4.1.2 Score differences between preceptors and teaching hospital nurses

This study found that preceptors had significantly higher total scores on the General Attitudes toward Artificial Intelligence Scale (GAAIS), Artificial Intelligence Literacy Scale (AILS), and Artificial Intelligence Self-Efficacy Scale (AISES) compared to non-preceptors, and nurses in teaching hospitals scored significantly higher than those in non-teaching hospitals (*p* < 0.05).

The higher scores among preceptors may be attributed to their teaching responsibilities, which require mastering AI technologies, such as intelligent monitoring systems or data analysis tools, thereby enhancing their AI literacy and positive attitudes (AILS and GAAIS). Additionally, practical experience and feedback gained during teaching may bolster their confidence in using AI (AISES). Bandura’s (32) self-efficacy theory posits that mastery experiences and vicarious experiences (e.g., guiding others) significantly enhance self-efficacy. The higher scores of teaching hospital nurses reflect the technological and educational advantages of their environment. Teaching hospitals, often affiliated with medical schools, are equipped with advanced AI technologies (e.g., intelligent diagnostic systems or robotic-assisted nursing) and offer more training opportunities. These conditions likely increase nurses’ understanding and familiarity with AI (AILS), improve their attitudes (GAAIS), and enhance confidence through practice (AISES). Lambert et al. (30) found that teaching hospitals, with abundant resources and frequent technology exposure, exhibit higher AI acceptance among healthcare professionals, whereas non-teaching hospitals, constrained by limited resources, restrict nurses’ AI cognition. This aligns with Goldfarb et al.’s (33) perspective on the advantages of teaching hospitals in AI adoption. Practically, hospitals can leverage preceptors to promote AI knowledge and attitudes, and teaching hospitals should support non-teaching hospitals through training to bridge the gap.

#### 4.1.3 Influence of educational level

This study found that nurses with a master’s degree or above had significantly higher scores on the General Attitudes toward Artificial Intelligence Scale (GAAIS), Artificial Intelligence Literacy Scale (AILS), and Artificial Intelligence Self-Efficacy Scale (AISES) compared to those with an associate degree or below and those with a bachelor’s degree or undergraduate education (*p* < 0.05). No significant differences were observed between the associate degree or below group and the bachelor’s degree or undergraduate group.

The higher scores among nurses with a master’s degree or above may be attributed to higher education providing more AI-related knowledge and skills training, such as courses on data analysis or intelligent healthcare systems, which enhance AI literacy (AILS) and positive attitudes (GAAIS). Additionally, research or practical opportunities may bolster their confidence in using AI (AISES). Dumić-Čule et al. (34) study indicated that higher education significantly improves healthcare professionals’ AI cognition and capabilities, particularly in understanding and applying intelligent healthcare systems. The lack of significant differences between the associate degree or below and bachelor’s degree groups may be due to the limited AI-related content in basic nursing education, which fails to distinctly differentiate the AI cognition of these two groups. This aligns with the Technology Acceptance Model (TAM), which posits that educational level influences perceived ease of use and usefulness, thereby enhancing attitudes and capabilities (11). Practically, nursing education should strengthen AI-related curricula, particularly for nurses with bachelor’s degrees or below, to improve their AI literacy and confidence.

#### 4.1.4 Differences by work department

This study found significant differences in scores on the General Attitudes toward Artificial Intelligence Scale (GAAIS), Artificial Intelligence Literacy Scale (AILS), and Artificial Intelligence Self-Efficacy Scale (AISES) between pediatric nurses and nurses in non-inpatient wards (*p* < 0.05), while pairwise comparisons among other departments showed no significant differences.

The higher scores among pediatric nurses may be attributed to the greater demand for AI technologies in their work. Pediatrics frequently employs intelligent monitoring systems or disease prediction models to monitor children’s conditions, and the frequent use of these tools likely enhances nurses’ familiarity with AI (AILS) and positive attitudes (GAAIS). Additionally, hands-on experience may strengthen their confidence in using AI (AISES). Karaarslan et al.’s (35) study indicated that the high usage rate of AI tools in pediatric nursing significantly improves healthcare professionals’ technology acceptance and capabilities. Non-inpatient ward nurses (e.g., outpatient or community nurses) primarily engage in health education or basic nursing tasks, where AI integration is limited, resulting in lower scores. Other departments (e.g., internal medicine, surgery) showed no significant differences, possibly due to relatively balanced levels of AI application. This aligns with the Technology Acceptance Model (TAM), which posits that the frequency of technology exposure influences perceived usefulness and attitudes (11). Practically, hospitals should increase AI application opportunities in non-inpatient departments to enhance nurses’ AI literacy and confidence.

#### 4.1.5 Influence of regional differences

This study, after applying case weighting adjustments by province, found that Beijing nurses had significantly higher scores on the General Attitudes toward Artificial Intelligence Scale (GAAIS), Artificial Intelligence Literacy Scale (AILS), and Artificial Intelligence Self-Efficacy Scale (AISES) compared to Sichuan nurses, and Sichuan nurses scored significantly higher than Yunnan nurses (*p* < 0.05).

Regional differences may stem from disparities in economic development levels and the distribution of medical technology resources. As an economic hub, Beijing boasts advanced healthcare systems and abundant AI resources, such as intelligent diagnostic systems and robotic nursing, which increase nurses’ exposure opportunities, enhancing AI literacy (AILS) and positive attitudes (GAAIS). Training and practical experience further bolster their confidence (AISES). Sichuan, with a relatively developed economy, exhibits moderate technology integration, while Yunnan, due to resource scarcity, has limited AI applications and training, resulting in the lowest scores. Amin et al. (36) indicated that regional medical technology levels significantly influence nurses’ AI acceptance, with urban areas demonstrating higher capabilities and confidence due to concentrated resources and robust technical support. Similarly, Lambert et al. (30) noted that urban hospitals, with frequent AI tool usage, show significantly higher acceptance and technical capabilities among healthcare professionals, whereas rural areas exhibit lower acceptance due to technological disparities. This aligns with the Technology Acceptance Model (TAM), which posits that technology availability influences perceived usefulness and attitudes (11). Practically, policies should increase AI investment in underdeveloped regions like Yunnan, providing equipment and training support. Remote education or collaborations with advanced hospitals in regions like Beijing can establish technology-sharing mechanisms, reducing regional disparities and promoting balanced AI application.

#### 4.1.6 Influence of training experience

This study found that nurses who received self-study or professional training had significantly higher Artificial Intelligence Literacy Scale (AILS) scores compared to those without any training (*p* < 0.05), with no significant differences between the self-study and professional training groups. For Artificial Intelligence Self-Efficacy Scale (AISES) scores, the professional training group scored significantly higher than the group with related thematic training or self-study, which in turn scored significantly higher than the no-training group, with all pairwise comparisons showing statistical significance (*p* < 0.05).

The improvement in AILS scores through training indicates that both self-study and professional training effectively enhance AI knowledge, such as skills in using intelligent healthcare tools. The lack of significant differences between self-study and professional training may be because both provide core knowledge, but professional training further enhances AISES through hands-on practice and guidance. Bandura (32) noted that mastery experiences, such as those gained through practical training, directly strengthen individuals’ confidence in their abilities via successful experiences. Amin et al. (36) found that professional training significantly boosts nurses’ confidence in AI through structured learning and feedback mechanisms, as evidenced by higher self-efficacy and usage intention, while self-study yields lesser effects. The no-training group’s lowest scores reflect the absence of exposure as a barrier to improving AI capabilities. Practically, hospitals should promote professional training to enhance nurses’ AI literacy and confidence while supporting self-study as a supplement to ensure all nurses have access to AI knowledge.

### 4.2 Correlation of AI general attitudes, AI literacy, and AI self-efficacy

This study found significant positive correlations among the total scores of the General Attitudes toward Artificial Intelligence Scale (GAAIS), Artificial Intelligence Literacy Scale (AILS), and Artificial Intelligence Self-Efficacy Scale (AISES), with correlation coefficients of GAAIS with AILS (r = 0.549, *p* < 0.001), GAAIS with AISES (r = 0.567, *p* < 0.001), and AILS with AISES (r = 0.684, *p* < 0.001). Additionally, AI usage intention showed significant positive correlations with the total scores of GAAIS, AILS, and AISES (*p* < 0.001).

These positive correlations suggest that the three factors form a mutually reinforcing positive cycle. Positive AI attitudes (GAAIS) may stimulate nurses’ motivation to learn, such as proactively exploring intelligent diagnostics or robotic nursing technologies, thereby enhancing AI literacy (AILS). Higher literacy, by increasing understanding and mastery of AI, further strengthens confidence in its use (AISES). Conversely, increased confidence and knowledge may reinforce positive evaluations of AI, creating a virtuous cycle. The high correlation between AILS and AISES (r = 0.684) is particularly notable, indicating that knowledge accumulation serves as the foundation for confidence. Wang and Chuang (28) found that AI self-efficacy is significantly positively correlated with literacy and attitudes, with knowledge and confidence jointly driving technology acceptance. Amin et al. (36) further noted that nurses’ positive attitudes toward AI are closely linked to their technical capabilities, supporting the positive cycle mechanism observed in this study. This aligns with the Technology Acceptance Model (TAM), which posits that perceived usefulness (attitudes) and ease of use (literacy) influence behavioral intention (11). The positive correlations of AI usage intention with all three factors suggest that positive attitudes, robust knowledge, and high confidence collectively promote nurses’ acceptance of AI. Practically, hospitals can enhance nurses’ literacy and self-efficacy through AI training while improving attitudes through promotional efforts, fostering a positive cycle to strengthen AI usage intention, which will facilitate the widespread application of AI in nursing.

### 4.3 Mediating roles of AI self-efficacy and AI general attitudes in the relationship between AI literacy and usage intention

This study, through structural equation modeling (SEM), revealed that AI self-efficacy (AISES) and general attitudes toward AI (GAAIS) play significant mediating roles between AI literacy (AILS) and AI usage intention (TODO). The mediation effects were as follows: AILS → AISES → TODO (0.207, accounting for 21.41% of the total effect), AILS → AISES → GAAIS → TODO (0.082, 8.48%), and AILS → GAAIS → TODO (0.141, 14.58%), with all paths significant at *p* < 0.001. The direct effect (AILS → TODO) was 0.295 (30.51%), and the total effect was 0.967, 95% CI [0.828–1.143].

Compared with findings from systematic and scoping reviews, this study’s effect sizes offer new insights. O’Connor et al.’s (4) systematic review indicates that nurses’ self-efficacy and positive attitudes are key drivers of AI adoption, but it does not provide specific mediation effect sizes. Similarly, Ng et al.’s (2) scoping review emphasizes that insufficient AI literacy and low technology acceptance limit AI application in nursing, with self-efficacy and attitudes playing significant roles in promoting adoption intentions. This study’s mediation effects (AISES: 21.41%; GAAIS: 14.58%) align with the qualitative conclusions of these reviews, quantifying the strength of AI literacy’s influence through confidence and attitudes. In contrast, Kwak et al. (37) reported a mediation effect of AI literacy on nursing students’ usage intentions through self-efficacy (path coefficients approximately 0.15–0.20), slightly lower than this study’s AISES path (0.207). This difference may stem from this study’s focus on Chinese nurses, whose unfamiliarity with AI likely amplifies the role of self-efficacy.

AI literacy indirectly promotes usage intentions by enhancing nurses’ confidence (AISES) and positive attitudes (GAAIS). The mediation effect of AISES is the strongest (21.41%), with path coefficients (AILS → AISES = 0.728, AISES → TODO = 0.172) indicating that AI literacy significantly boosts self-efficacy, which in turn drives usage intentions. This may be because AI knowledge (e.g., skills in intelligent monitoring systems or data analysis) enables nurses to feel confident in handling AI tasks, aligning with Bandura’s self-efficacy theory (32), which posits that mastery experiences (e.g., technical training) influence behavior through confidence. The mediation effect of GAAIS (14.58%) and the secondary path (8.48%) show that AI literacy improves attitudes (AILS → GAAIS = 0.343), which subsequently motivates usage (GAAIS → TODO = 0.261). The dual mediation path (AILS → AISES → GAAIS → TODO) has the smallest effect (8.48%), possibly due to the heterogeneous impact of attitudes on usage intentions across different regions (e.g., Sichuan and Yunnan vs. Beijing). Amin et al. (36) found that nurses’ positive attitudes and confidence significantly promote AI usage intentions, particularly in contexts where training enhances confidence. Wang and Chuang (28) highlighted the central role of self-efficacy in AI acceptance but did not deeply explore the interaction between attitudes and confidence. This study addresses this gap by elucidating the dual mediation path.

Contextual heterogeneity further explains the uniqueness of this study’ s findings. O′ Connor et al. (4) noted that the roles of self-efficacy and attitudes vary across cultural backgrounds and work environments, with Western nurses potentially exhibiting stronger direct effects (AILS → TODO) due to greater technological resources and widespread training. In contrast, Chinese nurses face regional disparities: limited technological resources in Sichuan and Yunnan may weaken the direct effect of AI literacy (30.51%), making AISES (21.41%) a more critical mediating pathway, as confidence compensates for reduced technology exposure. Conversely, Beijing’ s advanced technological environment may enhance the role of GAAIS (14.58%), as nurses are more likely to develop positive attitudes. This aligns with lower trust in new technologies in Chinese culture, where training should prioritize boosting confidence (2). Secinaro et al. (38) support that training and experience with AI tools enhance healthcare professionals’ acceptance, particularly in resource-limited regions. These findings are consistent with the Technology Acceptance Model (TAM), which posits that perceived ease of use (literacy) and attitudes drive behavioral intentions (11).

Practically, this study underscores the central role of AISES and recommends that hospitals implement structured training to enhance AI literacy and self-efficacy. In Sichuan and Yunnan, training should focus on foundational AI skills (e.g., operating intelligent devices) to bolster nurses’ confidence in AI tasks. In Beijing, promotional efforts and case studies can improve AI attitudes, facilitating technology adoption. Policymakers should account for regional resource disparities and develop tiered training strategies to support the widespread application of AI in nursing practice, ultimately improving care quality and patient outcomes.

## 5 Practical implications and policy recommendations

This study elucidated the mechanism by which AI literacy (AILS) influences nurses’ AI usage intention through AI self-efficacy (AISES) and general attitudes toward AI (GAAIS), providing significant insights for nursing practice and policy. The results indicate that AISES (21.41%) and GAAIS (14.58%) significantly facilitate usage intention as mediators, with the direct effect (30.51%) underscoring the critical role of literacy.

Practically, hospitals should design structured AI training programs, such as those focusing on intelligent monitoring systems and data analysis skills, to enhance nurses’ AI literacy and self-efficacy. Given that only 14.24% of nurses have received AI training, priority should be given to non-teaching hospitals and lower-level hospitals to bridge the technological gap. Preceptors, who scored higher, can serve as pioneers in AI promotion, disseminating knowledge and positive attitudes through teaching. Amin et al. (36) emphasized that training significantly boosts nurses’ confidence and acceptance. At the policy level, the government should address regional disparities (e.g., Beijing outperforming Yunnan) by providing subsidies or remote education to support AI resources and technology in underdeveloped regions. Secinaro et al. (38) suggested that policies should balance technology allocation to elevate overall healthcare standards. Additionally, hospitals can improve nurses’ attitudes toward AI through promotional activities, fostering a positive cycle of literacy, confidence, and attitudes to enhance usage intention. This will promote the widespread adoption of AI in nursing, ultimately improving patient care quality.

## 6 Limitations and future directions

Although this study provides empirical support for the application of AI in nursing, it has certain limitations. First, the sample was limited to public hospitals in Beijing, Sichuan, and Yunnan, and convenience sampling was employed, which may restrict the generalizability of the results to the national level or other healthcare institutions. Second, reliance on self-reported questionnaires to measure AI literacy, attitudes, and self-efficacy may be subject to social desirability bias, failing to reflect actual usage behavior. Additionally, the cross-sectional design cannot establish causality, and the SEM model fit was adequate (*χ*^2^/df = 4.581, RMSEA = 0.0824) but did not reach the ideal level (*χ*^2^/df < 3), possibly influenced by the large sample size (*n =* 878).

Future research could employ random sampling to expand the sample scope, covering more regions and hospital types to enhance representativeness. Incorporating objective indicators (e.g., frequency of AI tool usage) to validate self-reported results would help reduce bias. Longitudinal designs or intervention experiments could be used to track the dynamic impact of training on AI literacy and usage intention, clarifying causal relationships. Furthermore, optimizing the model structure by including variables such as organizational support or technology availability could refine the formation mechanism of AI usage intention. These improvements will help refine the theoretical framework and promote the application of AI in nursing practice.

## Data Availability

The data analyzed in this study is subject to the following licenses/restrictions: given the sensitive nature of the data, the de-identified dataset and surveys are not publicly accessible. However, reasonable requests for data access may be considered upon discussion with the research team. Requests to access these datasets should be directed to XZ, 1623131468@qq.com.

## Footnotes

^1^ https://www.wjx.cn/
